# Split k-mer analysis compared to cgMLST and SNP-based core genome analysis for detecting transmission of vancomycin-resistant enterococci: results from routine outbreak analyses across different hospitals and hospitals networks in Berlin, Germany

**DOI:** 10.1099/mgen.0.000937

**Published:** 2023-01-30

**Authors:** Friederike Maechler, Anna Weber, Oliver Schwengers, Frank Schwab, Luisa Denkel, Michael Behnke, Petra Gastmeier, Axel Kola

**Affiliations:** ^1^​ Institute of Hygiene and Environmental Medicine, Charité – Universitätsmedizin Berlin, Berlin, Germany; ^2^​ Bioinformatics and Systems Biology, Justus Liebig University Giessen, Giessen, Germany

**Keywords:** cgMLST, outbreak, split k-mer, SNP, Vancomycin-resistant Enterococcus faecium

## Abstract

The increase of Vancomycin-resistant *Enterococcus faecium* (VREfm) in recent years has been partially attributed to the rise of specific clonal lineages, which have been identified throughout Germany. To date, there is no gold standard for the interpretation of genomic data for outbreak analyses. New genomic approaches such as split k-mer analysis (SKA) could support cluster attribution for routine outbreak investigation. The aim of this project was to investigate frequent clonal lineages of VREfm identified during suspected outbreaks across different hospitals, and to compare genomic approaches including SKA in routine outbreak investigation. We used routine outbreak laboratory data from seven hospitals and three different hospital networks in Berlin, Germany. Short-read libraries were sequenced on the Illumina MiSeq system. We determined clusters using the published *Enterococcus faecium *-cgMLST scheme (threshold ≤20 alleles), and assigned sequence and complex types (ST, CT), using the Ridom SeqSphere+ software. For each cluster as determined by cgMLST, we used pairwise core-genome SNP-analysis and SKA at thresholds of ten and seven SNPs, respectively, to further distinguish cgMLST clusters. In order to investigate clinical relevance, we analysed to what extent epidemiological linkage backed the clusters determined with different genomic approaches. Between 2014 and 2021, we sequenced 693 VREfm strains, and 644 (93 %) were associated within cgMLST clusters. More than 74 % (*n*=475) of the strains belonged to the six largest cgMLST clusters, comprising ST117, ST78 and ST80. All six clusters were detected across several years and hospitals without apparent epidemiological links. Core SNP analysis identified 44 clusters with a median cluster size of three isolates (IQR 2–7, min-max 2–63), as well as 197 singletons (41.4 % of 475 isolates). SKA identified 67 clusters with a median cluster size of two isolates (IQR 2–4, min-max 2–19), and 261 singletons (54.9 % of 475 isolates). Of the isolate pairs attributed to clusters, 7 % (*n*=3064/45 596) of pairs in clusters determined by standard cgMLST, 15 % (*n*=1222/8500) of pairs in core SNP-clusters and 51 % (*n*=942/1880) of pairs in SKA-clusters showed epidemiological linkage. The proportion of epidemiological linkage differed between sequence types. For VREfm, the discriminative ability of the widely used cgMLST based approach at ≤20 alleles difference was insufficient to rule out hospital outbreaks without further analytical methods. Cluster assignment guided by core genome SNP analysis and the reference free SKA was more discriminative and correlated better with obvious epidemiological linkage, at least recently published thresholds (ten and seven SNPs, respectively) and for frequent STs. Besides higher overall discriminative power, the whole-genome approach implemented in SKA is also easier and faster to conduct and requires less computational resources.

## Data Summary

All supporting data, code and protocols have been provided within the article or as supplementary data files. Eight supplementary figures and tables as well as the code used for analysis and figure generation are available with the online version of this article. Sequencing data are accessible in the National Centre for Biotechnology Information (NCBI) database, BioProject ID PRJNA854814.

**Impact Statement**

Accurate and timely cluster attribution that is reproducible and comparable over time is essential for routine outbreak investigations. Recent introduction of target free methods promise better cluster resolution at lower costs in terms of hands-on time and computational resources. We compare cluster attribution for routine outbreak investigations using different genomic approaches at default settings (cgMLST) and at settings recently recommend by published literature (core SNP and SKA) [7]. While gene-based cgMLST was insufficient to draw distinctions between VREfm clusters from different hospitals and years, both SNP comparison of the core genomes and split k-mer analysis were able to discriminate between clusters. SKA showed even higher resolution and required less bioinformatics resources for frequent VREfm STs.

## Introduction

Although the prevalence rates of vancomycin-resistant *Enterococcus faecium* (VREfm) vary greatly between countries, the overall population-weighted prevalence in European countries increased from 10.5 % in 2015 up to 18.3 % in 2019 in European countries [1]. The increasing prevalence of VREfm in Germany [2] has been partly attributed to the rise of specific clonal lineages of hospital-associated isolates, which have been identified throughout the country. In recent years, the German National Reference Centre for Enterococci reported a marked increase of sequence type (ST)117 and ST80 [3]. The most frequent strains in German university hospitals on patient admission were ST117, ST80, ST203, and ST78, in that order [4]. At our hospital, Charité - Universitätsmedizin Berlin, we observed an increase of VREfm prevalence on admission from 1.10 to 2.59 per 100 admissions, and an increase of VREfm strain type ST117 complex type (CT)71 between 2008 and 2018 [5].

The development of whole genome sequencing (WGS) has meant immense progress for the elucidation of infection-epidemiological connections due to the high discrimination capacity of this method, and now represents the gold standard for bacterial strain typing [6], and there is still no ‘gold standard’ for the interpretation of WGS-data for epidemiological investigations [7]. To assess the genetic relatedness between bacterial isolates, different approaches are possible: on the one hand, gene-by-gene approaches compare the conserved nuclear genome (cgMLST), with or without adding the genes of the accessory genome (wgMLST). On the other hand, single-nucleotide polymorphisms (SNPs) of two bacterial genomes can be used for direct pairwise alignment and comparison [8].

SNP-typing has high computational requirements and is relatively time consuming [7, 9], whereas cgMLST-typing offers a standardized and stable nomenclature at low computational costs and bioinformatic prerequisites, especially when commercial software tools are used [8].

In contrast, target free, k-mer based methods such as the recently introduced target free split k-mer analysis (SKA) are currently not standard for transmission analyses [7, 10].

In consequence, the aim of this project was to investigate frequent clonal lineages of VREfm among suspected outbreaks of VREfm that were collected across different hospitals, hospital networks and over several years in a routine setting. In order to determine an appropriate method for molecular verification of suspected patient-to-patient transmission, we compared different approaches for genomic analyses including SKA.

## Methods

### Study population

We used screening and clinical patient samples as well as environmental specimens from devices and/or surfaces obtained during routine outbreak investigations from seven hospitals and three hospital networks in Berlin, Germany. One hospital network (Charité - Universitätsmedizin) comprised three separate hospital sites from a 3000-bed university hospital with a patient turnover of approximately 140 000 admissions per year that are located across the city and offers sequencing services to other local hospital sites. Two hospital sites from another hospital network were 640 and 670 bed tertiary care facilities, each admitting approximately 22000 patients per year. Two hospitals of 200 and 150 beds, respectively, with approximately 2500 and 8000 admissions per year constituted the third hospital network.

### Outbreak detection and infection prevention activities

Active surveillance screening is based on local infection control recommendations and usually comprised admission screening in high-risk wards such as hemato-oncological and solid organ transplant wards as well as screening of contact patients in case of suspected outbreaks or transmission chains. The three hospital sites from Charité - Universitätsmedizin have an automated outbreak detection system that combines patient movement and antimicrobial susceptibility data [11]. The four other hospital sites relied on the alertness of healthcare staff when evaluating culture results, their phenotypes, and their respective epidemiological relatedness to become suspicious of potential hospital outbreaks. Molecular analysis of genetic relatedness was only arranged for samples involved in suspected outbreaks. VREfm infection control management was in accordance with the German national guidelines [12].

### Microbiological cultures

We refer to an isolate as an *E. faecium* collected from a specific sample material that was cultured on chromID VRE agar plates (bioMérieux, Marcy-l´Étoile, France). Species identification and antimicrobial susceptibility testing was performed with Vitek2 System (bioMérieux, Marcy-l’Étoile, France) and MALDI-TOF MS (Bruker, Daltonics, Bremen, Germany).

### Whole genome sequencing

Isolation of genomic DNA from an overnight culture of VREfm was performed with the UltraClean Microbial DNA isolation kit (Qiagen, Hilden, Germany) following the manufacturer´s instructions. We used the Nextera XT DNA library preparation kit (Illumina Inc., San Diego, USA) and the MiSeq system (Illumina Inc., San Diego, USA) with 250-cycle paired-end chemistry according to the manufacturer’s instructions. Additionally, quality of raw sequence data was checked using FastQC at default settings [13].

### Bioinformatic analyses

1. For standard cgMLST analysis, quality-trimming, *de novo* assembly and gene-by-gene comparison were carried out with the SeqSphere+ software (Ridom GmbH, Muenster, Germany, version 8.3 at default settings) using the published 1423 gene *E. faecium* cgMLST task template (* E. faecium* reference genome NC_017022.1) [14, 15]. Raw sequencing reads were quality clipped with Trimmomatic [16] and *de novo* assembled with Velvet [17]. Sequence types (ST, https://pubmlst.org/), and core genome complex types (CT, https://www.cgmlst.org/ncs) were assigned using the Ridom SeqSphere+ software. CgMLST clusters were determined using pairwise allelic differences between isolates with a transmission cut-off of ≤20 alleles difference, which represents default settings. Accordingly, standard cgMLST-clusters comprised ≥2 isolates.

We did additional genomic analyses not included in the routine analysis workflow for each standard cgMLST-cluster separately, comprising the following:

2. We created ad hoc task template schemes using genomes from within the standard cgMLST-clusters for reference. Seed genomes were selected based on sequencing quality including a minimum average coverage of 48× and a percentage of good targets from the *E. faecium* task template scheme as determined by the SeqSphere+ software, range 94.0–99.4 %, and annotated with Prokka [18], and all other standard cgMLST-cluster isolates served as query genomes for cgMLST target definition with default settings. A complete list of reference genomes is available in the supplement (Supplementary Data S1, available in the online version of this article). Ad hoc cgMLST-cluster attribution and thresholds were the same as for standard cgMLST using SeqSphere+ software (Ridom GmbH, Muenster, Germany, version 8.3 at default settings).

3. We performed a core SNP analysis for each standard cgMLST-cluster using ASA³P [19] (https://github.com/oschwengers/asap, version 1.3.0 at default settings) for *de novo* assembly and annotation. Briefly, raw sequencing reads were quality clipped with Trimmomatic [16] and *de novo* assembled with SPAdes [20]. Contigs were rearranged using MeDuSa [21] and annotated with Prokka [18].

In order to compare the SNP based clustering with the standard cgMLST-clusters and ad hoc cgMLST-clusters, core genomes were aligned using Roary [22] (https://github.com/sanger-pathogens/Roary, version 3.13.0; settings: –e /create a multiFASTA alignment of core genes using PRANK). Pairwise SNP distances were calculated without masking of recombinations, and SNP distance matrices were built using snp-dist (https://github.com/tseemann/snp-dists, version snp-dists 0.8.2 at default settings). SciPy (https://github.com/scipy/scipy, version 1.8.0) was used for hierarchical single-linkage core SNP-clustering at a threshold of ten SNPs for putative inter-patient transmission based on published literature [7] that used intra-patient diversity to establish appropriate thresholds.

4. We conducted target free split k-mer analysis (SKA) and single-linkage clustering with the tool ‘SKA’ (https://github.com/simonrharris/SKA, version 1.0) developed by Harris *et al*. (2018 bioRxiv) [10], which we also refer to as SKA. We generated k-mer files (k=15) using the fasta subcommand with default settings. We calculated the pairwise distances and determined SKA-clusters at a threshold of seven SNPs if they met the required identity cutoff of at least 0.9, based on the same recent findings as for core SNP thresholds [7].

Phylogenetic trees were built using ASA³P [19] (https://github.com/oschwengers/asap version 1.3.0 at default settings), which uses consensus sequences created via BCFtools [23] (https://github.com/samtools/bcftools) and FastTreeMP [24](http://www.microbesonline.org/fasttree/), and the trees were visualized with iTOL, version 6.5 [25].

### Sensitivity analyses

As a sensitivity analysis, we performed SKA clustering without previous filtering based on standard cgMLST results to account for the different designs and scopes of the genomic approaches, and to ensure reproducibility in terms of the sample size, the selection of datasets and the underlying limitations of single linkage hierarchical clustering.

### Epidemiological analyses

We identified isolate pairs that were attributed to the same clusters for each genomic approach and categorized them according to their likely epidemiological linkage based on the location and the date of sample collection. Samples were collected a) from the same patient; b) from the same ward at the same time (same day); c) from the same ward at a different time (within 60 days); d) from the same hospital at the same time; e) from the same hospital at a different time (within 60 days); all other isolates were considered to have ‘no apparent epidemiologic linkage’.

In order to check for the epidemiological plausibility of cluster attribution, the number and percentage of cluster isolates that could be assigned to each of these categories were compared between the different genomic approaches.

### Cluster similarity and cluster variety by genomic approach

To investigate the diversity of cluster attribution, we determined intersecting cluster attributions and diversity indices for the different genomic approaches.

We used a simple measure for the diversity (cluster variety) in the examined population. In a group of N isolates analysed by genomic approach, a given isolate will be attributed to one of D distinct clusters, so we computed the simple diversity (SD1) as SD1=D/(N-1)*100 with 95 % exact binomial confidence intervals.

### Data collection and visualization

Patient-level data on patient admissions, day of sampling, hospital site, ward type and location, were collected through exports from the laboratory information system or through reports from the local hospitals’ infection control personnel.

Figures were generated in python 3.7.13 using matplotlib 3.5 and seaborn 0.11.2.

### Ethics

Approval by an institutional review board was not required, because VREfm isolates were collected and analysed during routine surveillance and infection control activities stipulated by National Healthcare Authorities.

## Results

### Standard cgMLST-cluster distribution

Between May 2014 and July 2021, infection control personnel required sequencing for 145 suspected outbreaks comprising 693 VREfm isolates from the seven hospitals and three hospital networks.

Of the isolates that underwent sequencing, routine cgMLST analyses attributed 93 % (*n*=644) to standard cgMLST-clusters of ≥2 isolates at a threshold of ≤20 alleles difference. In total, we identified 39 standard cgMLST-clusters with a median cluster size of four isolates (IQR 3–8, min-max 2–140). Of all isolates associated within standard cgMLST-clusters, 54 % (*n*=346) were part of ST117, 26 % (*n*=167) to ST80, 12 % (*n*=75) of ST78 and 5 % (*n*=35) of ST203. Other sequence types were identified in fewer than 1 % of standard cgMLST-cluster cases (four samples with unknown ST).

In total, 74 % (*n*=475) of all isolates were attributed to the six largest standard cgMLST-clusters, which we analysed with more detail. Of those, 93.8 % (*n*=441) were still part of original suspected outbreaks of ≥2 isolates.

**Fig. 1.**
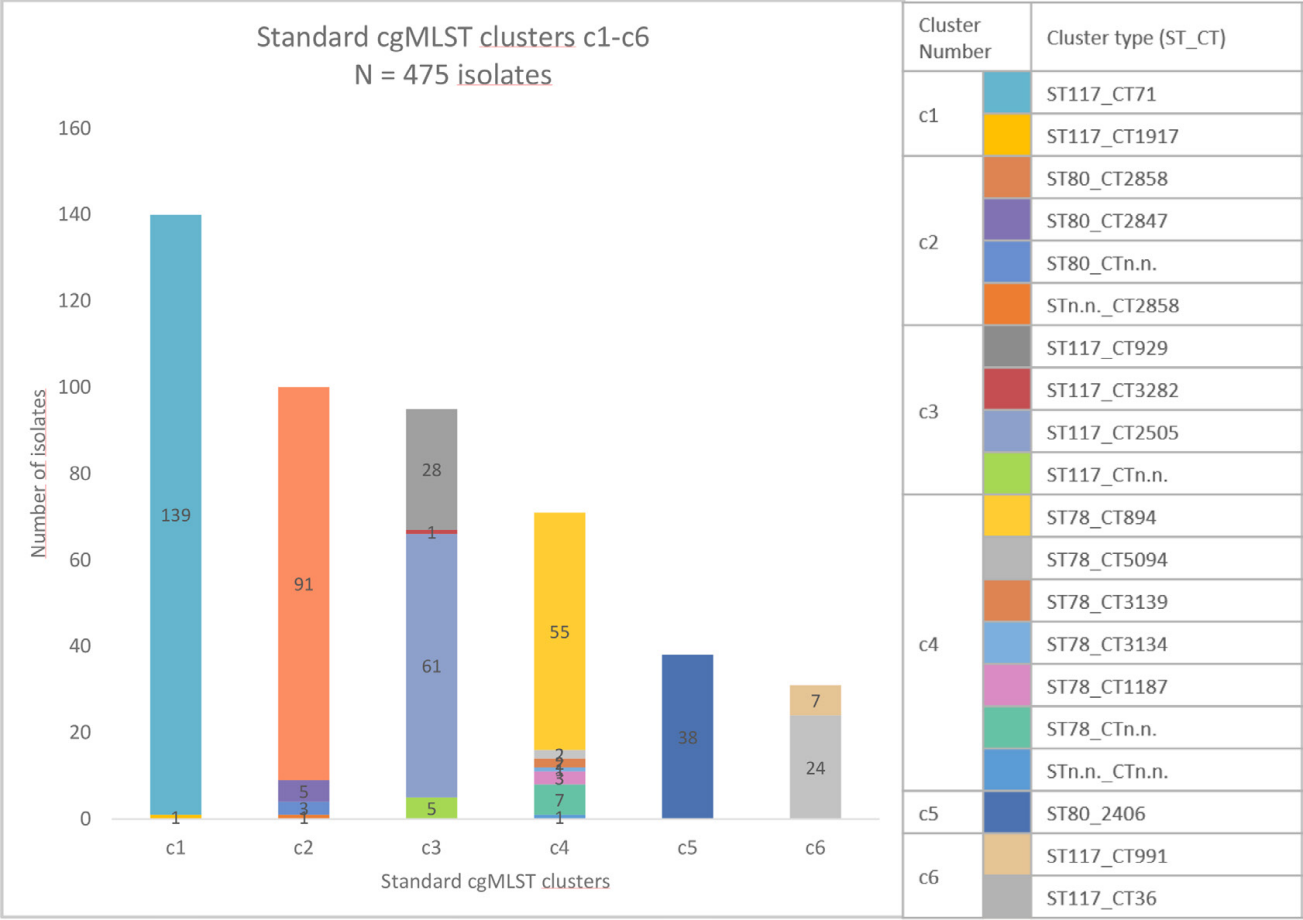
Overview of the six largest standard cgMLST-clusters (**c1-c6**). Colours represent cgMLST-complex types determined with Ridom SeqSphere+, numbers represent numbers of isolates.

In terms of standard cgMLST complex types, 29 % of the 475 isolates were determined as CT71 (*n*=139; ST117_CT71), 19 % as CT2858 (*n*=92; ST80_CT2858), 13 % as CT2505 (*n*=61; ST117_CT2505), 12 % as CT894 (*n*=55; ST78_CT894), 8 % as CT2406 (*n*=38; ST80_CT2406) and 6 % as CT929 (*n*=28; ST117_CT929). Other complex types were found in fewer than 5 % of isolates. Five of the six largest standard cgMLST-clusters comprised more than one complex type (Fig. 1).

Patient samples comprised 58 % (273/475) of screening samples from rectal swabs, nose swabs, throat swabs and stool samples, and 32 % (151/475) clinical cultures. Environmental samples comprised 10 % (49/475) of isolates. Eleven patients were sampled twice throughout the study period. Of those, the isolates from seven patients were determined as the same complex type and attributed to the same standard cgMLST-clusters (allele distance ≤2) both times. For the remaining four patients, we identified two different complex types and standard cgMLST-clusters (allele distance ≥20).

All six standard cgMLST-clusters stretched over ≥3 years, affected ≥5 hospitals and ≥2 hospital networks (Table 1, Supplementary Data S2 and S3). While standard cgMLST-cluster isolates from c1 and c6 were collected during the entire study period, c4 isolates were obtained between 2015 and 2021, c2 and c3 isolates between 2018 and 2021, and c5 isolates appeared only in 2021. Most of the isolates from the six standard cgMLST-clusters (93 %, 445/475) were collected between 2018 and 2021 (Table 1 and Supplementary Data S2).

**Table 1.** Characteristics of the six largest standard cgMLST-clusters and results of additional genomic approaches

Standard cgMLST		Suspected outbreaks	Ad hoc cgMLST		Core SNP			SKA			Van gene (N isolates)				Hospitals		Years
Name	*N* isolates	*N* clusters	*N* clusters	Median isolates per cluster (min-max)	*N* clusters	Median isolates per cluster (min-max)	SD1* (95 % CI)	*N* clusters	Median isolates per cluster (min-max)	SD1* (95 % CI)	A	B	A+B	Not found	*N* Hospital Networks	*N* Hospitals	*N*
**c1**	140	28	1	na (na-139)	10	3 (2-54)	0.46 (0.404–0.610)	18	2.5 [1–6, 8]	0.525 (0.342–0.547)	--	135	3	2	3	6	6
**c2**	100	21	2	50 (4-96)	4	6 (2-63)	0.263 (0.150–0.361)	15	2 [1–6, 8, 9]	0.626 (0.481–0.721)	94	3	--	3	3	6	3
**c3**	95	20	6	3,5 (2-78)	11	4 [1–6, 8–29]	0.404 (0.266–0.510)	12	2 [1–6, 8–19]	0.638 (0.489–0.735)	86	3	--	6	3	5	4
**c4**	71	8	8	3 [1–6, 8–42]	12	3 [1–6, 8–13]	0.486 (0.318–0.608)	12	2.5 [1–6, 8–16]	0.514 (0.344–0.636)	71	--	--	--	3	5	4
**c5**	38	5	2	19 [1–6, 8–36]	4	6.5 [2–6, 8–11]	0.378 (0.174–0.552)	5	5 [1–6, 8–10]	0.297 (0.116–0.470)	38	--	--	--	2	5	2
**c6**	31	6	2	15.5 [6, 8–24]	3	3 [1–6, 8–13]	0.5 (0.248–0.687)	4	2 [1–6, 8–12]	0.433 (0.195–0.626)	30	--	--	1	2	5	6

* SD1 are simple measures for the diversity (cluster variety) with 95 % exact binomial confidence intervals

### Cluster attribution by different genomic approaches

**Fig. 2.**
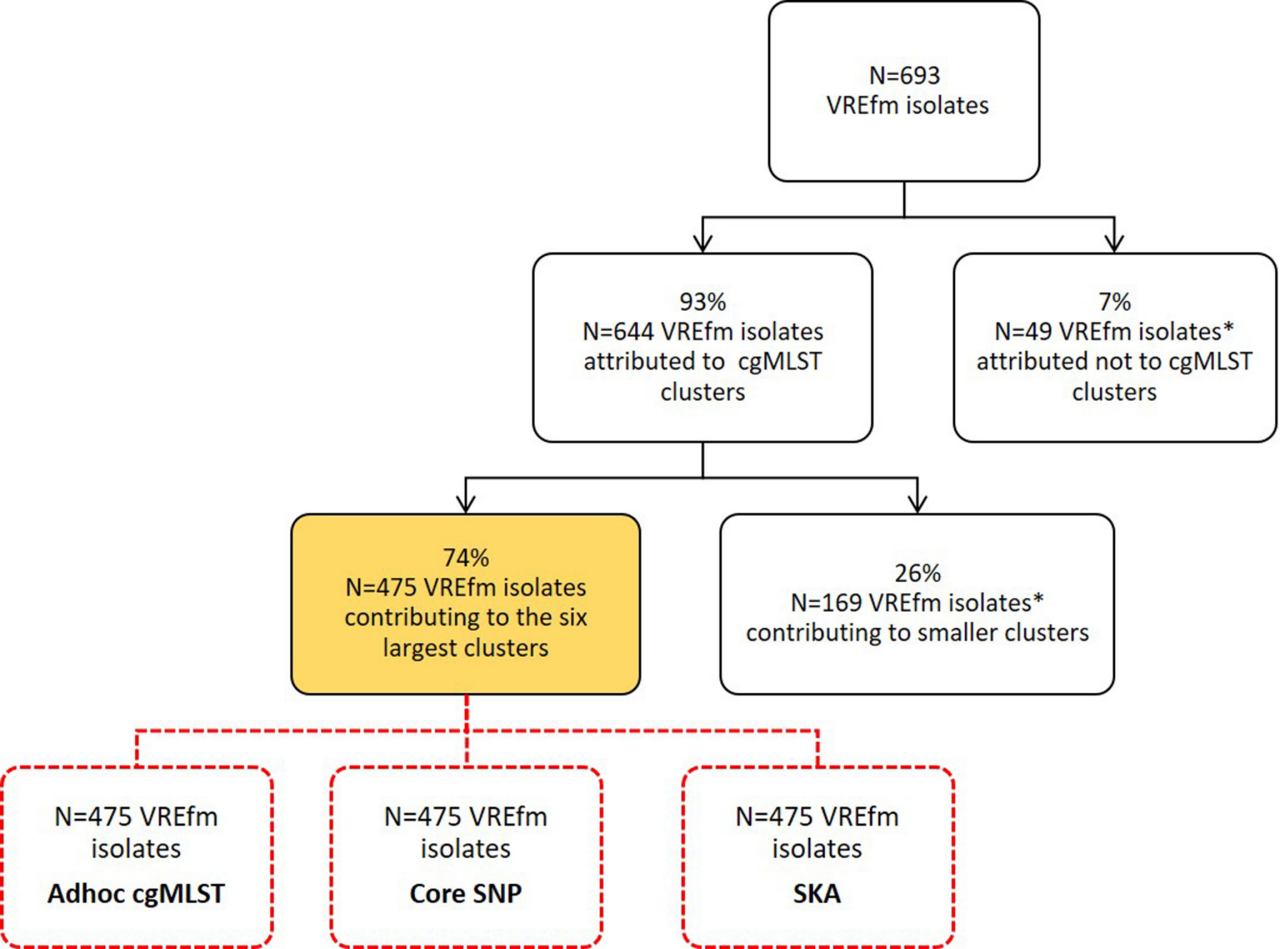
Overview of study samples used within different genomic approaches.

Fig. 2 shows the genomic approaches used in the different datasets.

Ad hoc cgMLST analysis identified 21 clusters with a median cluster size of four isolates (IQR 2–16.5, min-max 2–139), and three singletons. Standard cgMLST-clusters c3 und c4 were broken down into smaller ad hoc cgMLST-clusters, but still resulted in relatively large cluster sizes comprising up to 42 (c3) and 78 (c4) isolates. For most standard cgMLST-clusters (4/6), ad hoc cgMLST did not draw further distinctions.

Core SNP analysis identified 44 clusters with a median cluster size of three isolates (IQR 2–7, min-max 2–63), as well as 197 singletons (41.4 % of 475 isolates). Median core genome SNP distances within standard cgMLST-clusters were 40.5 SNPs (IQR 24–156) for c1, 25 SNPs (IQR 16–40) for c2, 70 SNPs (IQR 37–244) for c3, 411 SNPs (IQR 100–497) for c4, 34 SNPs (IQR 24–41) for c5 and 54 SNPs (IQR 27–839) for c6.

SKA identified 67 clusters with a median cluster size of two isolates (IQR 2–4, min-max 2–19), and 261 singletons (54.9 % of 475 isolates). Median whole genome SKA SNP distances within standard cgMLST-clusters were 41.0 SNPs (IQR 26–60) for c1, 33.0 SNPs (IQR 23–51) for c2, 129 SNPs (IQR 69–272) for c3, 150 SNPs (IQR 91–240) for c4, 29 SNPs (IQR 20–36) for c5 and 25 SNPs (IQR 15–364) for c6.

Table 1 shows an overview of the cluster attributions per genomic approach and the number of isolates they encompassed, the van genes per standard cgMLST-cluster as well as the years, the hospitals and hospital networks the isolates were collected from. Supplementary Data S4 shows the ad hoc task target schemes determined with reference genomes from within the standard cgMLST-clusters and the number of targets they comprised.

**Fig. 3.**
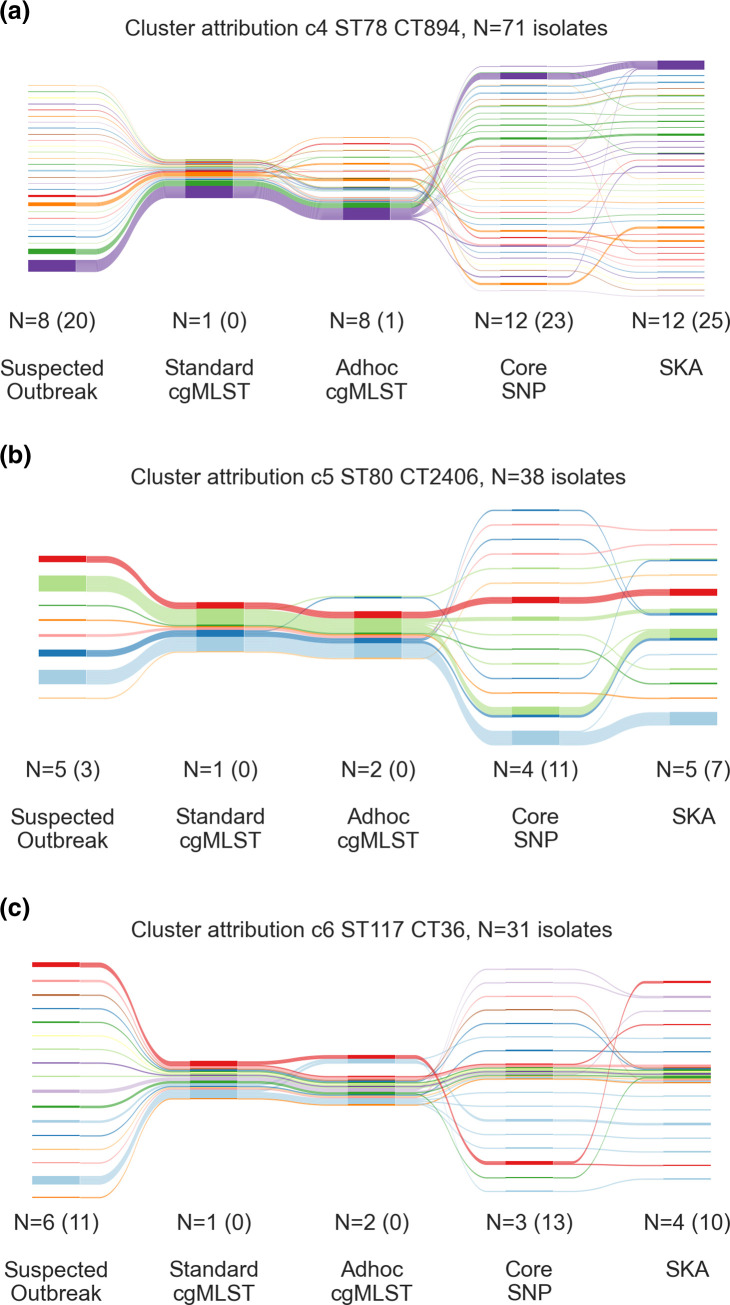
Relationship between standard cgMLST-clusters and clusters as determined by pairwise core genome SNP-analysis and SKA for three of the six largest cgMLST-clusters (**c4, c5 and c6**). Depicted are the suspected outbreaks which determined the colours (Suspected Outbreak) and the clusters the isolates were attributed to by each genomic approach; the column size represents the number of isolates. N are clusters, (**n**) are singletons.

Fig. 3 shows exemplary cluster attributions by the different genomic approaches for three of the six standard cgMLST-clusters. In all cases, the suspected outbreaks from different hospitals and hospital networks were all attributed to one single standard cgMLST-cluster. Ad hoc cgMLST did not draw further distinctions between the outbreaks from different sites. Core SNP analysis and SKA broke down the standard cgMLST-clusters into smaller clusters and singletons, which often correlated with the suspected outbreaks. The larger standard cgMLST-clusters c1-c3 showed the same pattern (Supplementary Data S5).

### Similarity of cluster attribution between SKA and Core SNP analysis

In total, 119 isolates (25%) were identified as singletons both with Core SNP and with SKA.

Of the isolates attributed to clusters of ≥2 isolates with one of the two methods, 21.9 % (104/475) were attributed to the same clusters using both methods. In total, 39.8 % of isolates (189/475) were Core SNP-clusters that were broken down into smaller SKA-clusters, while 11.4 % of isolates (54/475) were smaller Core SNP-clusters that merged into bigger SKA-clusters. The remaining 2.9 % (9/475) were broken into non-matching clusters between the two methods.

Diversity indices for SKA-clusters and Core SNP-clusters were similar, except for c2, which showed higher diversity for SKA (Table 1)

### Sensitivity analysis

The primary analyses grouped the 475 isolates based on the clusters established by standard cgMLST analysis (c1-c6), and then applied SKA for each standard cgMLST-cluster separately. For sensitivity analyses, we used SKA and single-linkage clustering on the entire set of 475 isolates without previous standard cgMLST. SKA-cluster attribution with and without previous standard cgMLST clustering matched 100 %.

### Epidemiological link of isolates associated within clusters based on the different genomic approaches

**Fig. 4.**
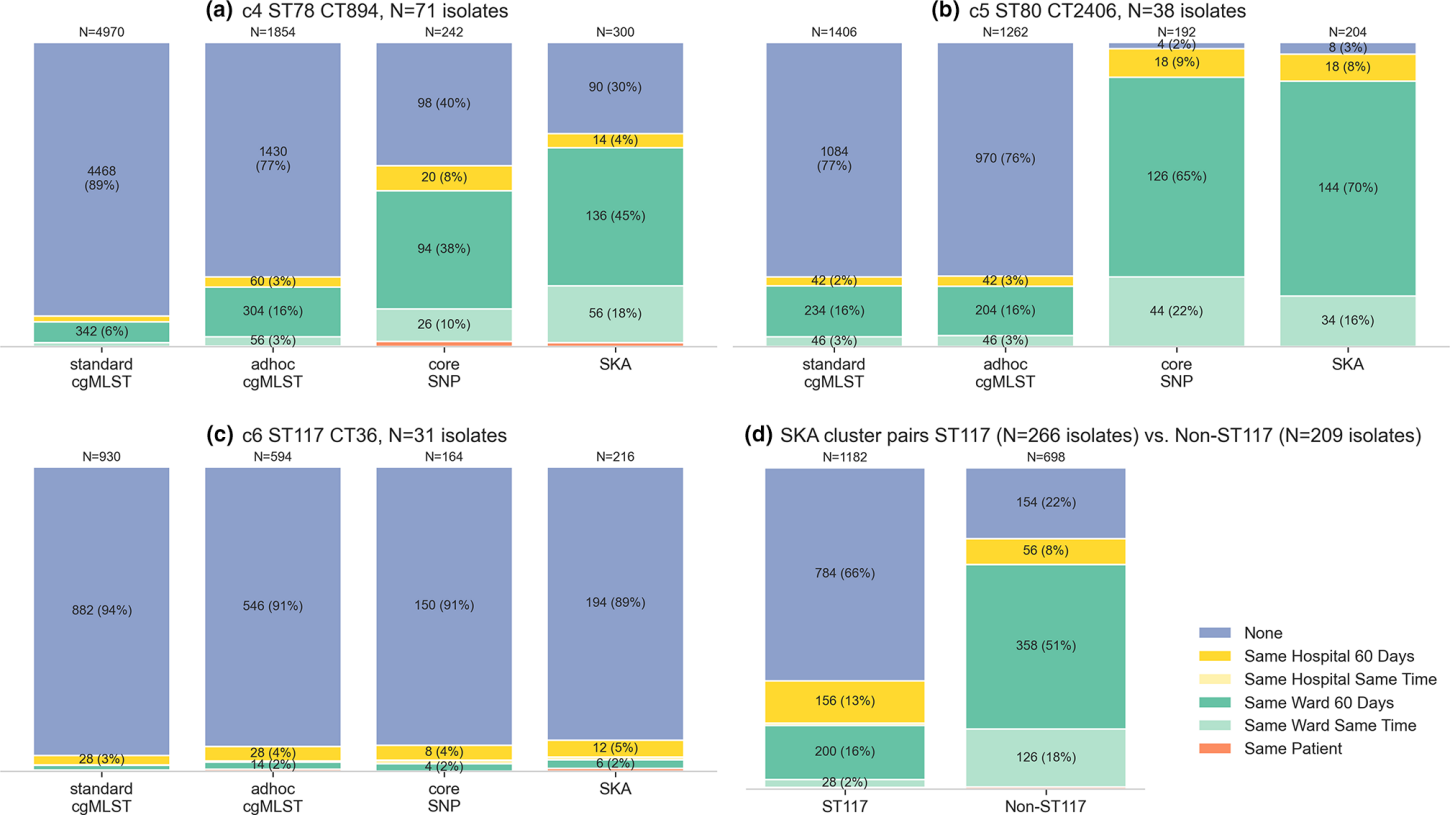
Number and proportion of pairwise epidemiological links for all isolate pairs attributed to clusters (a) c4, (b) c5, (c) c6 by genomic approach and (d) for ST117 clusters (c1, c3 and c6) vs. Non-ST117 clusters (c2, c4 and c5) attributed to clusters by SKA. Depicted are isolate pairs attributed to clusters using standard cgMLST, and among those the pairs attributed to any cluster using the alternative genomic approaches. N are isolate pairs. Colours represent categories of epidemiological linkage.

In order to compare the epidemiological links, we determined the number of isolate pairs that were attributed to clusters for each genomic approach. Among all 112 575 potential isolate pairs between the 475 isolates, 45 352 were attributed to standard cgMLST-clusters, 37 832 to ad hoc cgMLST-clusters, 8352 to core SNP-clusters, and 1862 to SKA-clusters. Of those isolate pairs attributed to clusters, 7 % of pairs in clusters determined by standard cgMLST, 8 % of pairs in clusters ad hoc cgMLST-clusters, 15 % of pairs in core SNP-clusters and 51 % of pairs in SKA-clusters were linked according to the epidemiological categories (see Fig. 4). When analysing the isolate pairs in clusters for each standard cgMLST-cluster separately, we saw varying degrees of epidemiological linkage. For isolate pairs from c2 (ST80), c4 (ST78) and c5 (ST80), 70–98 % of cluster attribution by core SNP and SKA was backed up by epidemiological linkage. Isolate pairs for c1, c3 and c6 were all determined as ST117 and showed epidemiological linkage for 11–50 % of core SNP- and SKA-clusters (see Fig. 3 and Supplementary Data S6).

### Phylogenies

**Fig. 5.**
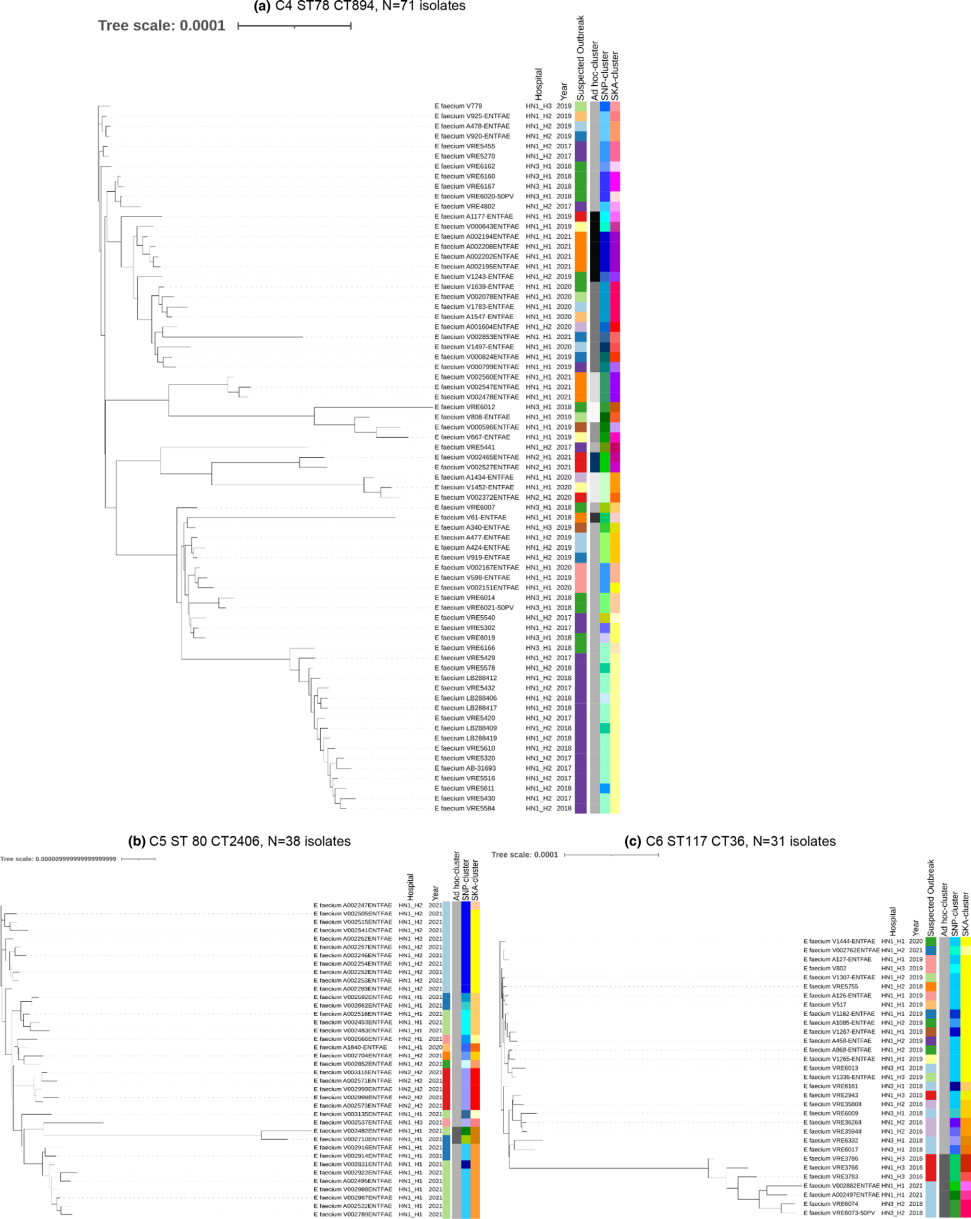
Core SNP-Phylogenetic approximately maximum-likelihood trees of standard cgMLST clusters (a) c4 (*n*=71), (b) c5 (*n*=38) and (c) c6 (*n*=31) built within ASA³P (FastTreeMP) and visualized with iTOL. Colours in the heatmap indicate the different clusters of the respective genomic approaches.

Fig. 5 shows phylogenic trees and the respective cluster attribution by genomic approach as well as collection year and site for standard cgMLST-clusters c4, c5 and c6. The supplementary appendix provides phylogenetic trees for the larger standard cgMLST-clusters c1, c2 and c3 (Supplementary Data S7).

## Discussion

To our knowledge, this is the first routine use and confirmation of SKA as an accurate and rapid tool for routine VREfm outbreak investigations after its recent first introduction by Higgs and colleagues [7].

Though the published gene-based *E. faecium* cgMLST typing schemes promised an equivalent discriminatory power to that of an SNP-based approach [14], the results obtained with this method for the widespread *E. faecium* types ST 78, ST80 ad ST 117 in the present study did not meet these expectations. Standard cgMLST was insufficient to confirm or rule out suspected VREfm transmission chains and outbreaks, because it was not possible to draw distinctions between isolates without epidemiological linkage based on the time and/or the location the samples were collected from.

Certain complex types such as ST117 CT71 have been identified in epidemiologically unrelated settings all over Germany [3–5]. Some of the complex types identified in Berlin were also found during outbreak investigations in Bavaria and the Rhine-Main area [26, 27]. In this project, most large standard cgMLST-clusters including but not limited to ST117 CT71 spanned over several years and across several hospitals and even hospital networks. Cross-institutional dominance of certain complex types seemed to be the rule rather than the exception.

However, just because transmission seems epidemiologically unlikely, genomic results should not be immediately dismissed. There may be transmission both inside the hospital and in the community that we could not account for, but patient-to-patient transmission chains across the entire country over a period of several years appear rather unlikely. For most of the isolates of this project, standard cgMLST and ad hoc cgMLST could not differentiate between the putative community spread without any epidemiological links on the one hand, and hospital outbreaks that could be targeted by local infection prevention activities on the other hand.

To make it more confusing and even misleading when inferring recent hospital transmission from the complex type attributions, several standard cgMLST-clusters comprised more than one complex type (ST_CT).

As reported by others, additional approaches were required to resolve the outbreaks [28, 29]. Both core SNP analysis as well as SKA were able to break down the standard cgMLST-clusters into smaller clusters that corresponded with local epidemiological data from the hospitals.

Core genome based approaches compare only a fraction of the entire genome. For species other than *E. faecium *, cgMLST approaches have been used widely and successfully during outbreak analyses [30, 31]. For VREfm, however, recent literature showed only limited ability to distinguish between cgMLST-clusters, regardless of the threshold settings [26, 27].

Reference choice and sample diversity have been reported to heavily affect clustering results [32]. In terms of sample diversity, a dynamic core genome-based approach over an extended period of time could have decreased the target genes for analysis and thus overestimated transmission events [32]. We used the published 1423 target core genome scheme [14] throughout the 7 years of analysis, so the number of target genes was fixed and did not change over time. This core genome size is small compared with the recently published core genome size of 1926 genes [33] for the hospital adapted clade A1 of clinical isolates [34]. We did not see a better ability to distinguish between clusters across hospitals and years when adapting the standard cgMLST approach by using reference genomes from within the standard cgMLST-clusters and thus increasing the number of target genes to an average of around 2100 genes for ad hoc cgMLST analysis.

Moreover, including the accessory genome into the cgMLST approaches did not fundamentally change the differentiation between clusters (data not shown).

In contrast, core SNP analysis and SKA had more discriminative power and correlated better with suspected outbreaks and epidemiological categories. SKA as a reference free approach encompassing the entire genome resulted in more singletons and more but smaller clusters than core SNP analysis. Considering the extensively mobilizable and expanding pangenome of *E. faecium *, this seems plausible [33, 35]. For the hospital adapted clade A1 of clinical isolates [34], the role of plasmid gain contributing to cluster emergence and diversification has been described previously, with more than 40 % of genes identified on plasmids and more than a third of cluster-defining genes found on plasmids in at least one isolate [33]. Accounting for those genes, which are typically not included in core gene approaches, appears therefore essential in outbreak investigations.

Regarding the concordance of cluster attribution between core SNP analysis and SKA, almost half of the isolates were attributed to identical clusters or were equally identified as singletons using both SKA and core SNP analysis. Most of the remaining isolates were core SNP-clusters that were broken down into smaller SKA-clusters, which seems intuitive, because a core genome approach should be less discriminative when compared to an approach that encompasses the entire genome, especially in a species with a reportedly high plasticity of the genome [36, 37].

Besides determining the approaches with the best accuracy for inferring genomic relatedness, there is also the question of feasibility in terms of computational capacity and bioinformatics hands-on time during routine outbreak investigations.

SKA was faster than core SNP and much easier to use, because it does not require *de novo* assembly, annotation and core gene alignment prior to clustering. However, SKA was specifically designed to analyse closely related genomes, such as those from an outbreak or transmission chain – at least at default settings. As a consequence, it seems necessary to first narrow down the sample diversity to more closely related isolates by a more traditional genomic approach such as MLST or even cgMLST in a first step, followed by SKA [7]. However, we confirmed the robustness of SKA results in a sensitivity analysis, and cluster identification with and without standard cgMLST prior to SKA showed 100 % consistence and thus remarkable stability across the different sequence and complex types.

Of note, the standard cgMLST-clusters that showed less potential for better resolution using additional methods were all ST117. In comparison, there may be more undetected clonal spread and a more stable genome. ST117 has been described as highly adapted for effective transmission and persistence in hospital settings because of genes coding for collagen adherence and biofilm formation such as espfm, efa, afm, acm and scm [26, 38].

Currently, there is no reliable genomic gold standard for inferring transmissions. Routine hospital outbreak investigations should be balanced to accurately identify between-patient transmissions while considering their epidemiological likelihood. For practical purposes, genomic approaches should reflect recent transmission rather than phylogenetic relatedness through a relatively stable core genome over time, to incite swift and adapted infection control measures. Thus, for short-term investigations such as routine outbreak analyses, SKA as a rapid whole-genome approach seems to be the logical choice, at least as an additional method.

The downgrading of hospital outbreaks through appropriate molecular analyses to guide infection control management could have immediate and palpable financial impacts, if ruling out transmissions avoids the blocking of hospital beds or the closure of entire wards for new admission. The costs of a hospital bed per day range between approximately 150€ (non-critical care beds) and 1500€ (critical care beds) [39]. Considering the expenditure for blocked hospital beds on the one hand and the costs of whole genome sequencing to rule out hospital outbreaks on the other hand, quickly shows the potential for savings.

There are several limitations to our analysis.

User specific cut-offs and definitions as well as biases in the underlying data complicate comparison. We did not have enough VREfm isolates collected from the same patients to determine core SNP- and/or SKA-cluster thresholds based on intra-patient vs. inter-patient SNP distances. We used cut-offs recently published for the same species and a similar research question across several hospitals and years that were derived from intra-patient diversity and that also used data for assessing the level of intra-patient genomic diversity, who had previously sequenced multiple colonies from individual cultures [7, 40]. For cgMLST, we also used thresholds suggested by recent literature (≤3, ≤10, ≤15 alleles, respectively) [41, 42] that were not part of our main analysis. These cut-offs did not fundamentally change our results, although the number of clusters was similar for the three alleles cgMLST-approach and SKA. However, especially for the larger standard cgMLST-clusters, the maximum number of isolates comprised in single clusters with the three alleles threshold included approximately half of all isolates (Supplementary Data, Table S9), and SKA results matched better with the suspected outbreaks.

Because this was an analysis of routine laboratory data and sequencing results based on suspected outbreaks, not a comprehensive analysis of all potential outbreaks within the hospitals. An important limitation of outbreak detection across most German hospitals is a lack of funding for sequencing. Moreover, most of the wards did not employ active surveillance screening for carriage of VREfm on admission, so we could not rule out that patients were already colonized on admission.

We may have missed potential patient contacts within hospitals, because we did not have complete patient movement data including stays on wards without collection of samples. Movement of healthcare staff, patients or even equipment within, between and across hospitals is often hard to account for. Moreover, movement data could contain errors and may miss other patient contacts such as shared equipment, e.g. during invasive or radiologic procedures. The combination of genomic data and detailed patient-contact modelling could certainly assist future outbreak investigations [29].

We did not mask recombinations and could therefore have underestimated transmission events. Because masking of ancient recombination would reduce the size of the core genome without improving (or likely even worsening) the reliability of the inference of recent transmission events, recent publications recommended not masking recombinations for VREfm transmission analyses [7].

We did not specifically analyse potential horizontal transmission of mobile genetic elements as an additional driver of VREfm spread [43–45].

### Conclusions

In this study, we could show that allele-based cgMLST approaches with the commonly used threshold settings of ≤20 alleles difference were insufficient for transmission inference of VREfm from routine outbreak data. Core SNP-analysis and the reference free SKA at thresholds based on recent literature (seven and ten SNPs, respectively) had more discriminative power and correlated better with suspected local hospital outbreaks as well as systematic epidemiological categorizations. SKA had the highest correlation with both outbreak and epidemiological data at the lowest costs in terms of computational resources. Therefore, our findings endorse SKA as an easy to use method for molecular verification of suspected VREfm transmissions in clinical settings with results comparable to core SNP analysis, at least for frequent STs.

## Supplementary Data

Supplementary material 1
