# Supplementary material for: Split k-mer analysis compared to cgMLST and SNP-based core genome analysis for detecting transmission of vancomycin-resistant enterococci: results from routine outbreak analyses across different hospitals and hospitals networks in Berlin, Germany
Source: Microb Genom. 2023 Jan 30;9(1):mgen000937. doi: 10.1099/mgen.0.000937 (PMC9973845; doi:10.1099/mgen.0.000937)
Supplement: Supplementary material 1 [file mgen-9-937-s001.pdf]

Appendix (Supplementary Data)

S1: Table of reference genomes used in the study.

Cluster name	Number samples	Seed Genome for adhoc cgMLST (SeqSphere)
c1	140	V2776
c2	100	V2179
c3	95	V2783
c4	71	V2078
c5	38	A1840
c6	31	VRE5755

S2: Figure shows the distribution of the six standard cgMLST clusters throughout the years (2015-2021).

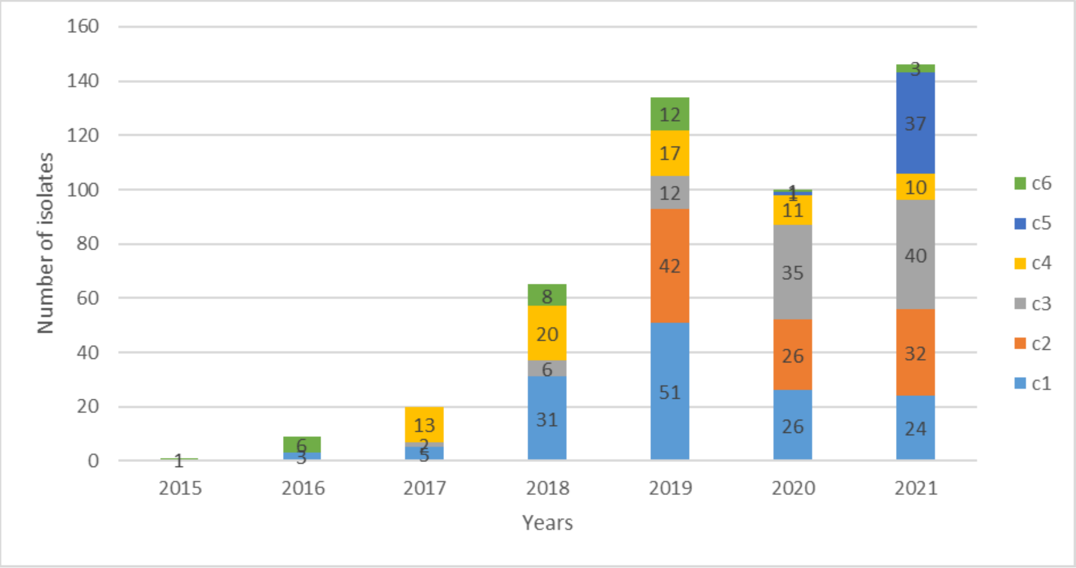

S3: Figure shows the cluster distribution of the six standard cgMLST clusters (c1-c6) across the different hospital networks (HT) and hospitals (H).

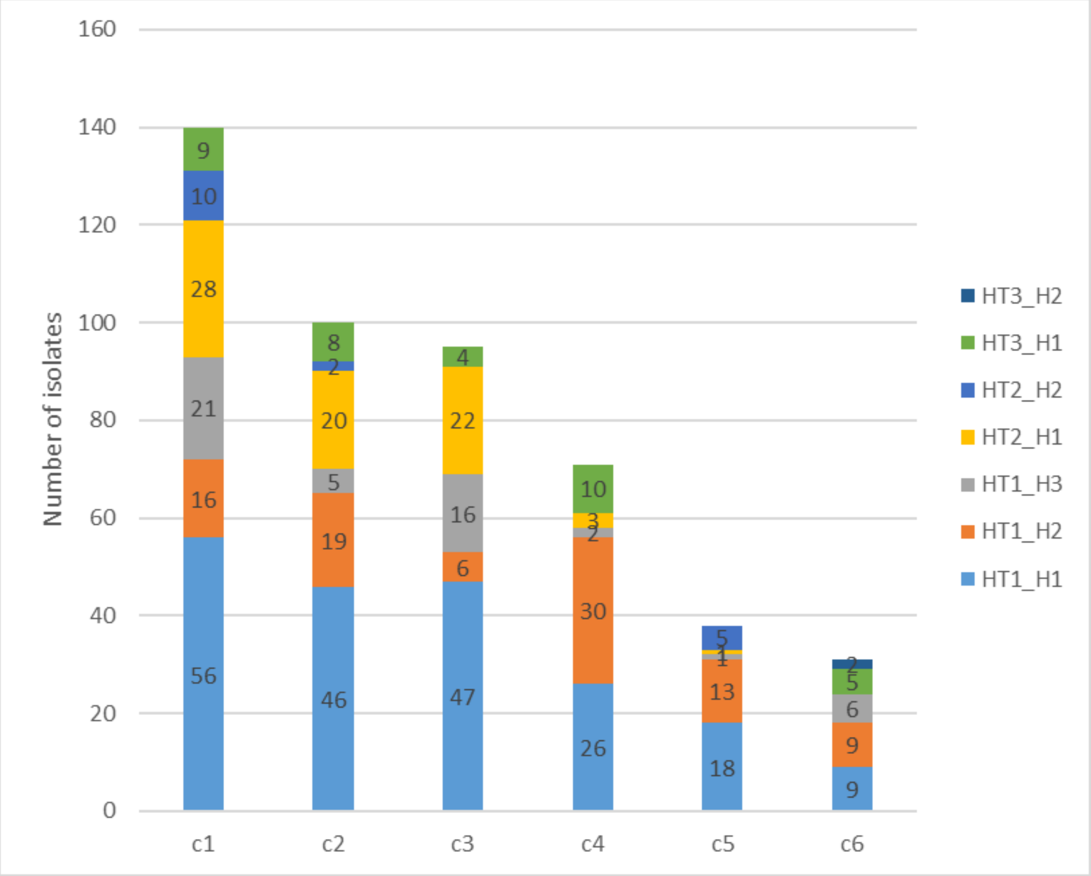

S4: Average distance (min - max) in standard cgMLST clusters and adhoc cgMLST clusters (c1-c6).

Cluster name	Standard cgMLST	Adhoc cgMLST	Number targets adhoc cgMLST scheme
c1	9.757 (0 - 36)	13.4 (0 - 45)	1989
c2	10.614 (0 - 35)	15.252 (0 - 47)	2004
c3	16.198 (0 - 40)	27.89 (0 - 72)	2068
c4	18.793 (0 - 46)	32.109 (0 - 72)	2095
c5	9.381 (0 - 22)	18.03 (0 - 41)	2544
c6	11.302 (0 - 29)	22.923 (0 - 65)	2409

S5: Relationship between cgMLST clusters and clusters as determined by pairwise core genome SNP-analysis and SKA for three of the six largest cgMLST clusters a) c1, b) c2, c) c3. N are clusters, (N) are singletons.

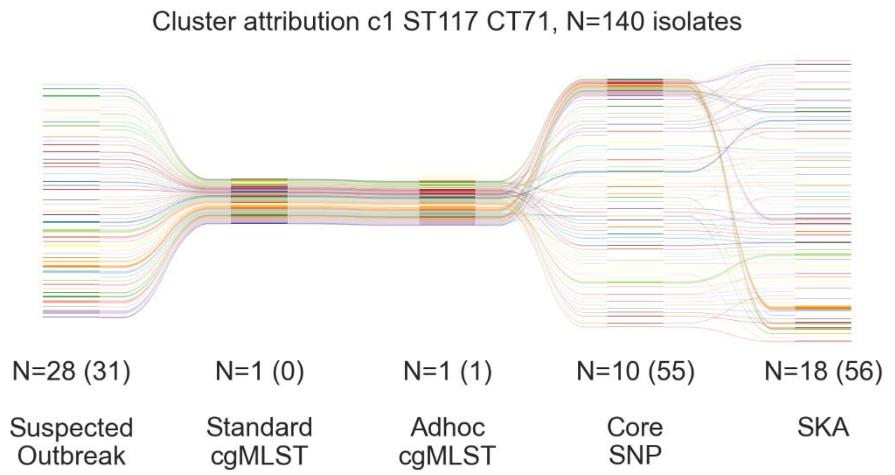

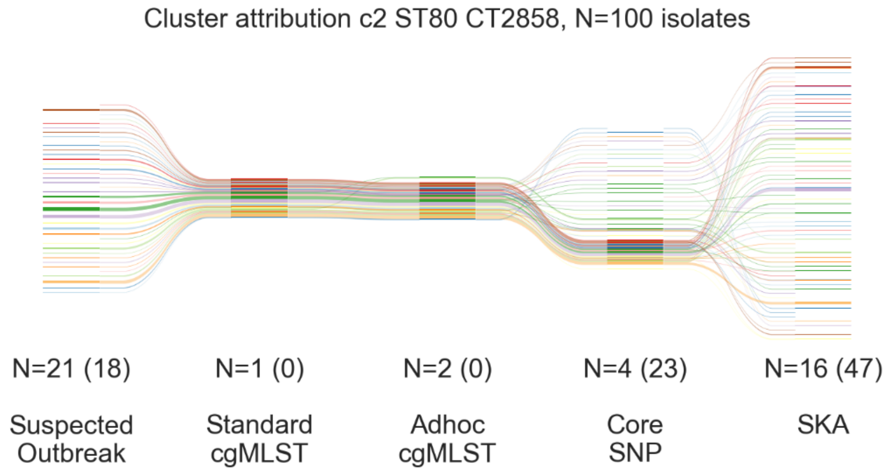

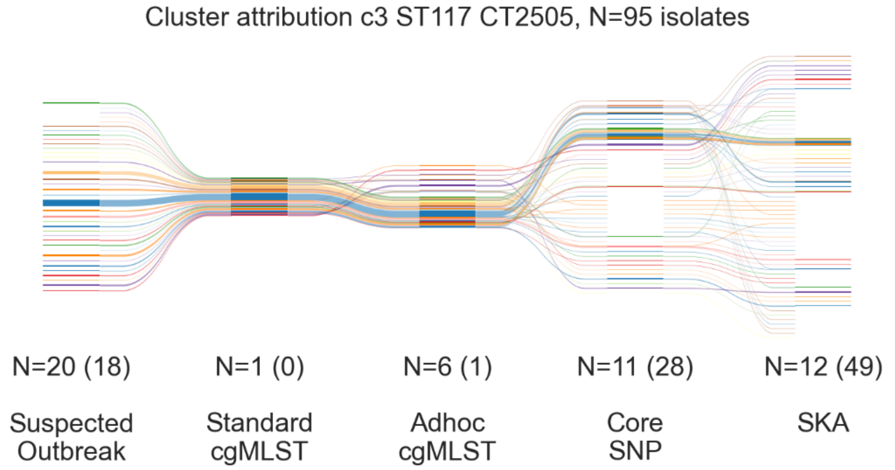

S6: Number and proportion of pairwise epidemiological links for all isolate pairs attributed to clusters a) c1, b) c2, c) c3 by genomic approach

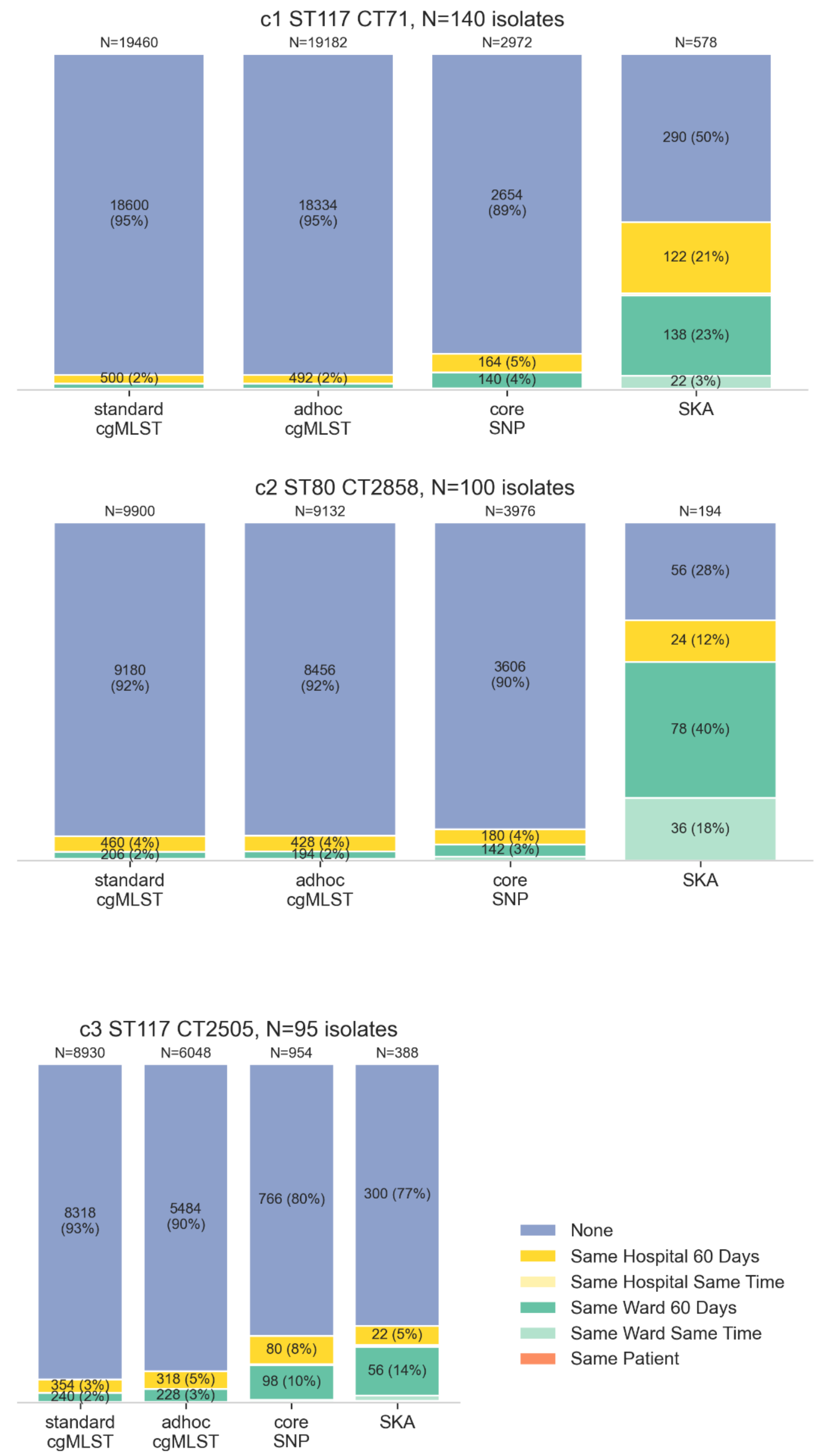

S7: SKA SNP distance heatmaps

c1

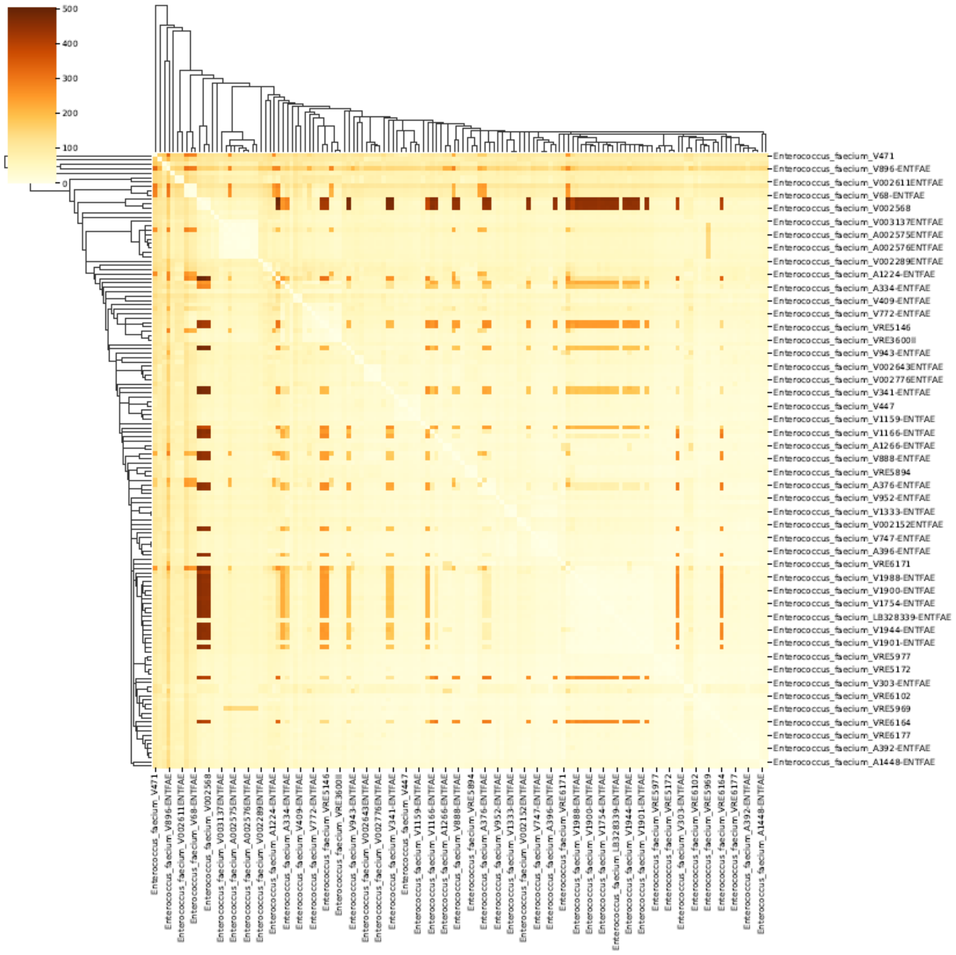

C2

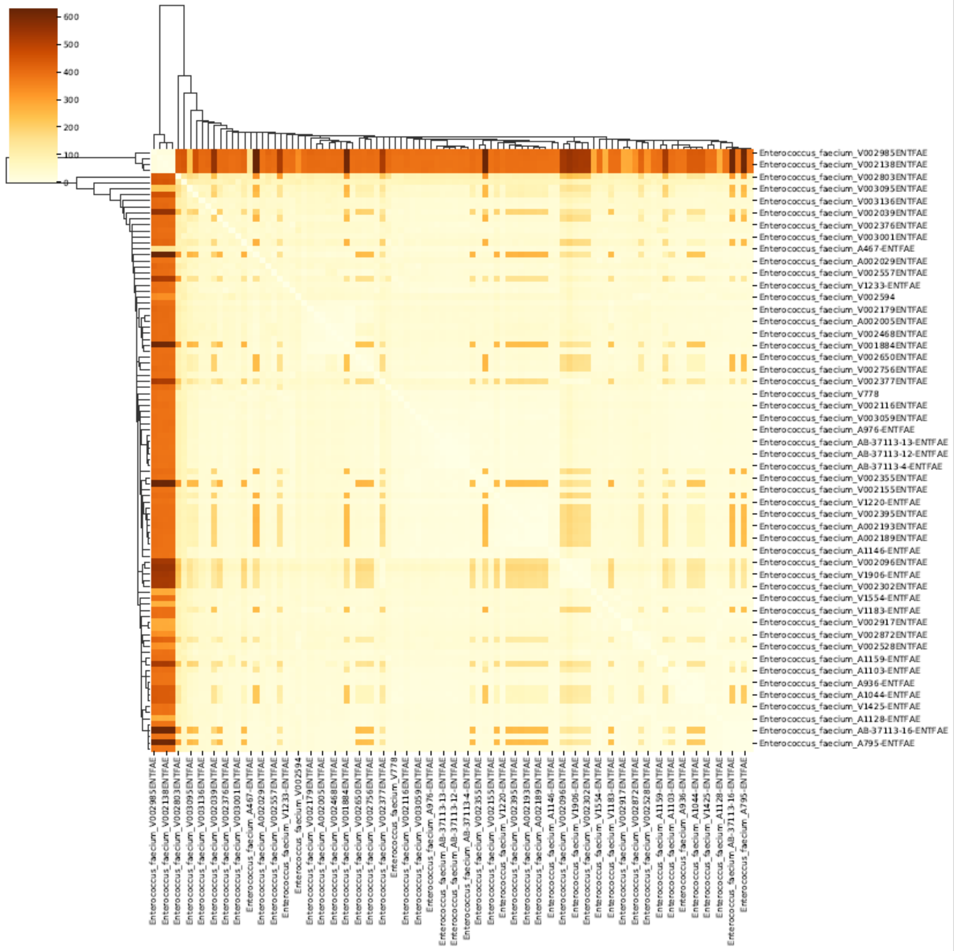
600
500
400
300
200
100
0
Enterococcus_faecium_V002985ENTFAE
Enterococcus_faecium_V002138ENTFAE
Enterococcus_faecium_V002803ENTFAE
Enterococcus_faecium_V003095ENTFAE
Enterococcus_faecium_V003136ENTFAE
Enterococcus_faecium_V002039ENTFAE
Enterococcus_faecium_V002376ENTFAE
Enterococcus_faecium_V003001ENTFAE
Enterococcus_faecium_A467-ENTFAE
Enterococcus_faecium_A002029ENTFAE
Enterococcus_faecium_V002557ENTFAE
Enterococcus_faecium_V1233-ENTFAE
Enterococcus_faecium_V002594
Enterococcus_faecium_V002179ENTFAE
Enterococcus_faecium_A002005ENTFAE
Enterococcus_faecium_V002468ENTFAE
Enterococcus_faecium_V001884ENTFAE
Enterococcus_faecium_V002650ENTFAE
Enterococcus_faecium_V002756ENTFAE
Enterococcus_faecium_V002377ENTFAE
Enterococcus_faecium_V778
Enterococcus_faecium_V002116ENTFAE
Enterococcus_faecium_V003059ENTFAE
Enterococcus_faecium_A976-ENTFAE
Enterococcus_faecium_AB-37113-13-ENTFAE
Enterococcus_faecium_AB-37113-12-ENTFAE
Enterococcus_faecium_AB-37113-4-ENTFAE
Enterococcus_faecium_V002355ENTFAE
Enterococcus_faecium_V002155ENTFAE
Enterococcus_faecium_V1220-ENTFAE
Enterococcus_faecium_V002395ENTFAE
Enterococcus_faecium_A002193ENTFAE
Enterococcus_faecium_A002189ENTFAE
Enterococcus_faecium_A1146-ENTFAE
Enterococcus_faecium_V002096ENTFAE
Enterococcus_faecium_V1906-ENTFAE
Enterococcus_faecium_V002302ENTFAE
Enterococcus_faecium_V1554-ENTFAE
Enterococcus_faecium_V1183-ENTFAE
Enterococcus_faecium_V002917ENTFAE
Enterococcus_faecium_V002872ENTFAE
Enterococcus_faecium_V002528ENTFAE
Enterococcus_faecium_A1159-ENTFAE
Enterococcus_faecium_A1103-ENTFAE
Enterococcus_faecium_A936-ENTFAE
Enterococcus_faecium_A1044-ENTFAE
Enterococcus_faecium_V1425-ENTFAE
Enterococcus_faecium_A1128-ENTFAE
Enterococcus_faecium_AB-37113-16-ENTFAE
Enterococcus_faecium_A795-ENTFAE

C3

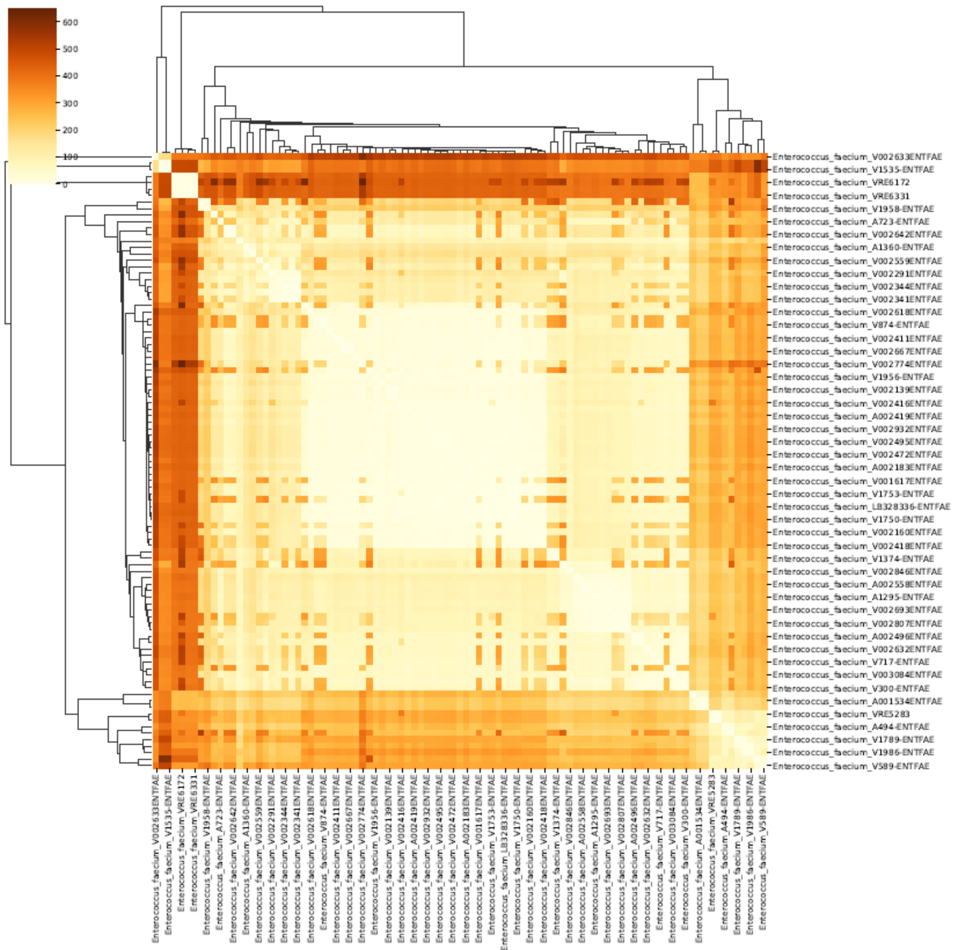
600
500
400
300
200
100
0
Enterococcus_faecium_V002633ENTFAE
Enterococcus_faecium_V1535-ENTFAE
Enterococcus_faecium_VRE6172
Enterococcus_faecium_VRE6331
Enterococcus_faecium_V1958-ENTFAE
Enterococcus_faecium_A723-ENTFAE
Enterococcus_faecium_V002642ENTFAE
Enterococcus_faecium_A1360-ENTFAE
Enterococcus_faecium_V002559ENTFAE
Enterococcus_faecium_V002291ENTFAE
Enterococcus_faecium_V002344ENTFAE
Enterococcus_faecium_V002341ENTFAE
Enterococcus_faecium_V002618ENTFAE
Enterococcus_faecium_V874-ENTFAE
Enterococcus_faecium_V002411ENTFAE
Enterococcus_faecium_V002667ENTFAE
Enterococcus_faecium_V002774ENTFAE
Enterococcus_faecium_V1956-ENTFAE
Enterococcus_faecium_V002139ENTFAE
Enterococcus_faecium_V002416ENTFAE
Enterococcus_faecium_A002419ENTFAE
Enterococcus_faecium_V002932ENTFAE
Enterococcus_faecium_V002495ENTFAE
Enterococcus_faecium_V002472ENTFAE
Enterococcus_faecium_A002183ENTFAE
Enterococcus_faecium_V001617ENTFAE
Enterococcus_faecium_V1753-ENTFAE
Enterococcus_faecium_LB328336-ENTFAE
Enterococcus_faecium_V1750-ENTFAE
Enterococcus_faecium_V002160ENTFAE
Enterococcus_faecium_V002418ENTFAE
Enterococcus_faecium_V1374-ENTFAE
Enterococcus_faecium_V002846ENTFAE
Enterococcus_faecium_A002558ENTFAE
Enterococcus_faecium_A1295-ENTFAE
Enterococcus_faecium_V002693ENTFAE
Enterococcus_faecium_V002807ENTFAE
Enterococcus_faecium_A002496ENTFAE
Enterococcus_faecium_V002632ENTFAE
Enterococcus_faecium_V717-ENTFAE
Enterococcus_faecium_V003084ENTFAE
Enterococcus_faecium_V300-ENTFAE
Enterococcus_faecium_A001534ENTFAE
Enterococcus_faecium_VRE5283
Enterococcus_faecium_A494-ENTFAE
Enterococcus_faecium_V1789-ENTFAE
Enterococcus_faecium_V1986-ENTFAE
Enterococcus_faecium_V589-ENTFAE

C4

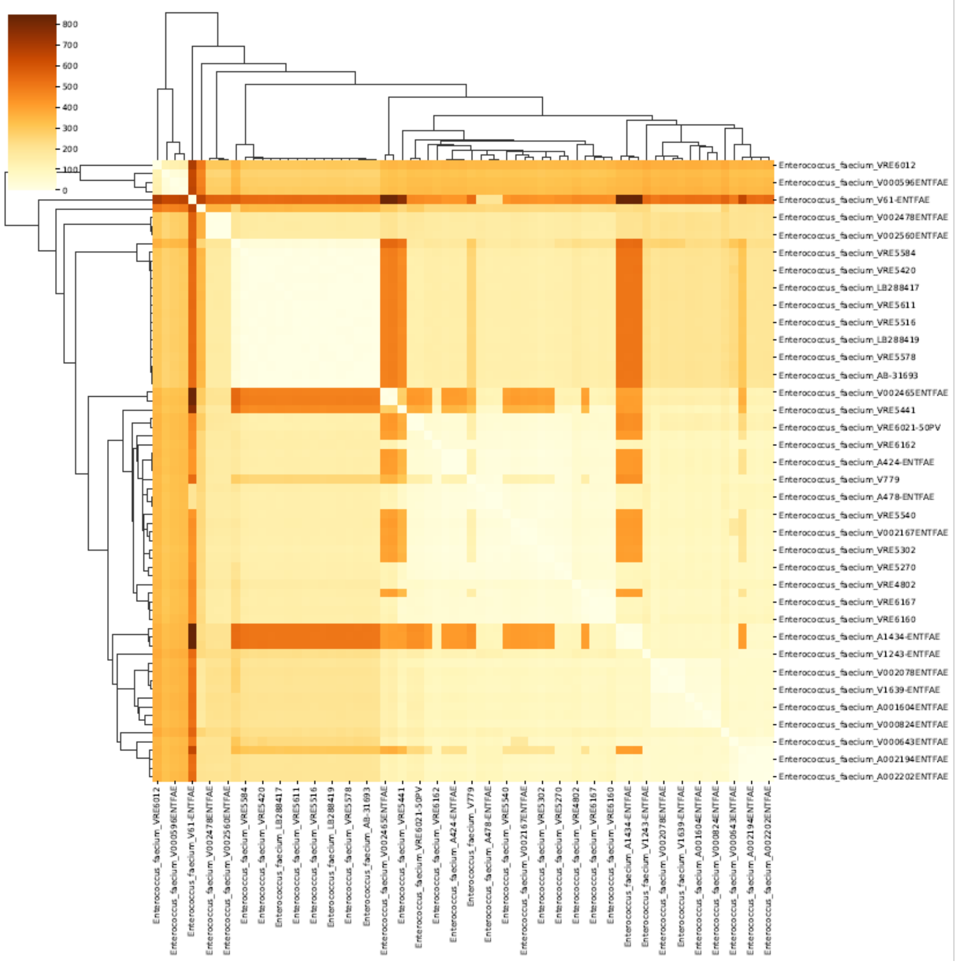

800
700
600
500
400
300
200
100
0
Enterococcus_faecium_VRE6012
Enterococcus_faecium_V000596ENTFAE
Enterococcus_faecium_V61-ENTFAE
Enterococcus_faecium_V002478ENTFAE
Enterococcus_faecium_V002560ENTFAE
Enterococcus_faecium_VRE5584
Enterococcus_faecium_VRE5420
Enterococcus_faecium_LB288417
Enterococcus_faecium_VRE5611
Enterococcus_faecium_VRE5516
Enterococcus_faecium_LB288419
Enterococcus_faecium_VRE5578
Enterococcus_faecium_AB-31693
Enterococcus_faecium_V002465ENTFAE
Enterococcus_faecium_VRE5441
Enterococcus_faecium_VRE6021-50PV
Enterococcus_faecium_VRE6162
Enterococcus_faecium_A424-ENTFAE
Enterococcus_faecium_V779
Enterococcus_faecium_A478-ENTFAE
Enterococcus_faecium_VRE5540
Enterococcus_faecium_V002167ENTFAE
Enterococcus_faecium_VRE5302
Enterococcus_faecium_VRE5270
Enterococcus_faecium_VRE4802
Enterococcus_faecium_VRE6167
Enterococcus_faecium_VRE6160
Enterococcus_faecium_A1434-ENTFAE
Enterococcus_faecium_V1243-ENTFAE
Enterococcus_faecium_V002078ENTFAE
Enterococcus_faecium_V1639-ENTFAE
Enterococcus_faecium_A001604ENTFAE
Enterococcus_faecium_V000824ENTFAE
Enterococcus_faecium_V000643ENTFAE
Enterococcus_faecium_A002194ENTFAE
Enterococcus_faecium_A002202ENTFAE

C5

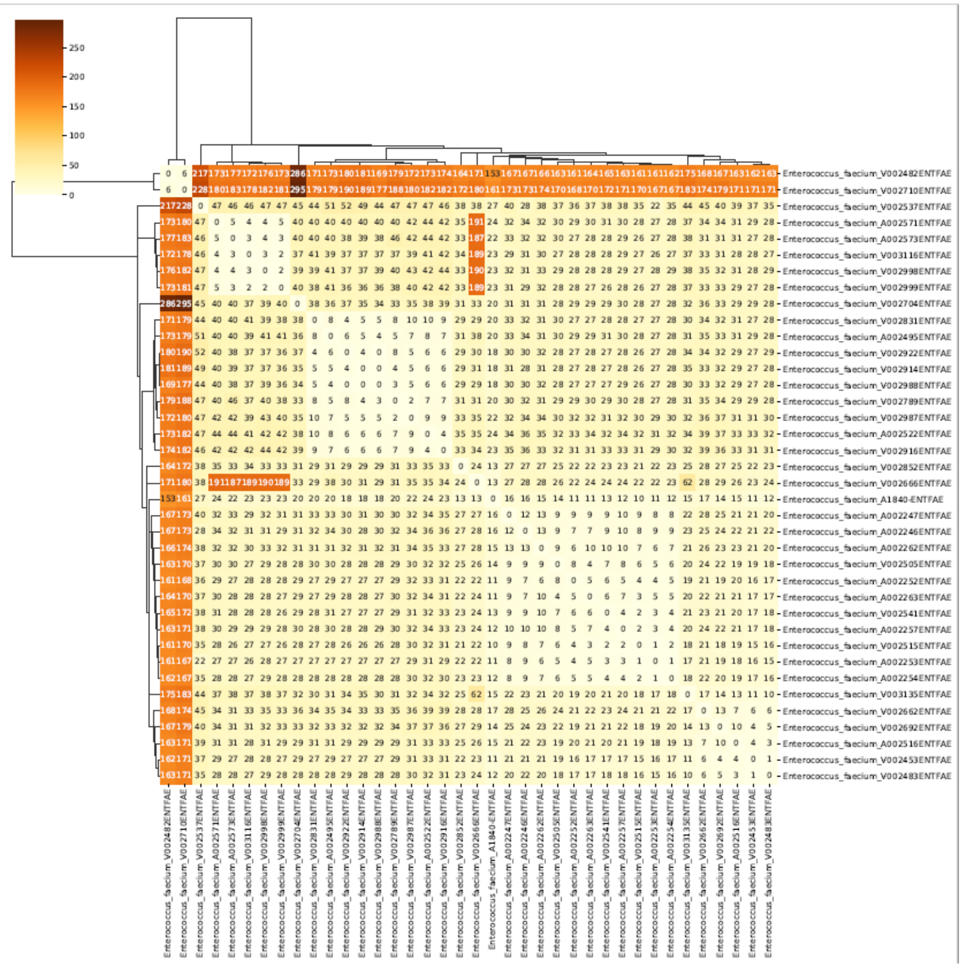
250
200
150
100
50
0
Enterococcus_faecium_V002482ENTFAE
Enterococcus_faecium_V002710ENTFAE
Enterococcus_faecium_V002537ENTFAE
Enterococcus_faecium_A002571ENTFAE
Enterococcus_faecium_A002573ENTFAE
Enterococcus_faecium_V003116ENTFAE
Enterococcus_faecium_V002998ENTFAE
Enterococcus_faecium_V002999ENTFAE
Enterococcus_faecium_V002704ENTFAE
Enterococcus_faecium_V002831ENTFAE
Enterococcus_faecium_A002495ENTFAE
Enterococcus_faecium_V002922ENTFAE
Enterococcus_faecium_V002914ENTFAE
Enterococcus_faecium_V002988ENTFAE
Enterococcus_faecium_V002789ENTFAE
Enterococcus_faecium_V002987ENTFAE
Enterococcus_faecium_A002522ENTFAE
Enterococcus_faecium_V002916ENTFAE
Enterococcus_faecium_V002852ENTFAE
Enterococcus_faecium_V002666ENTFAE
Enterococcus_faecium_A1840-ENTFAE
Enterococcus_faecium_A002247ENTFAE
Enterococcus_faecium_A002246ENTFAE
Enterococcus_faecium_A002262ENTFAE
Enterococcus_faecium_V002505ENTFAE
Enterococcus_faecium_A002252ENTFAE
Enterococcus_faecium_A002263ENTFAE
Enterococcus_faecium_V002541ENTFAE
Enterococcus_faecium_A002257ENTFAE
Enterococcus_faecium_V002515ENTFAE
Enterococcus_faecium_A002253ENTFAE
Enterococcus_faecium_A002254ENTFAE
Enterococcus_faecium_V003135ENTFAE
Enterococcus_faecium_V002662ENTFAE
Enterococcus_faecium_V002692ENTFAE
Enterococcus_faecium_A002516ENTFAE
Enterococcus_faecium_V002453ENTFAE
Enterococcus_faecium_V002483ENTFAE

C6

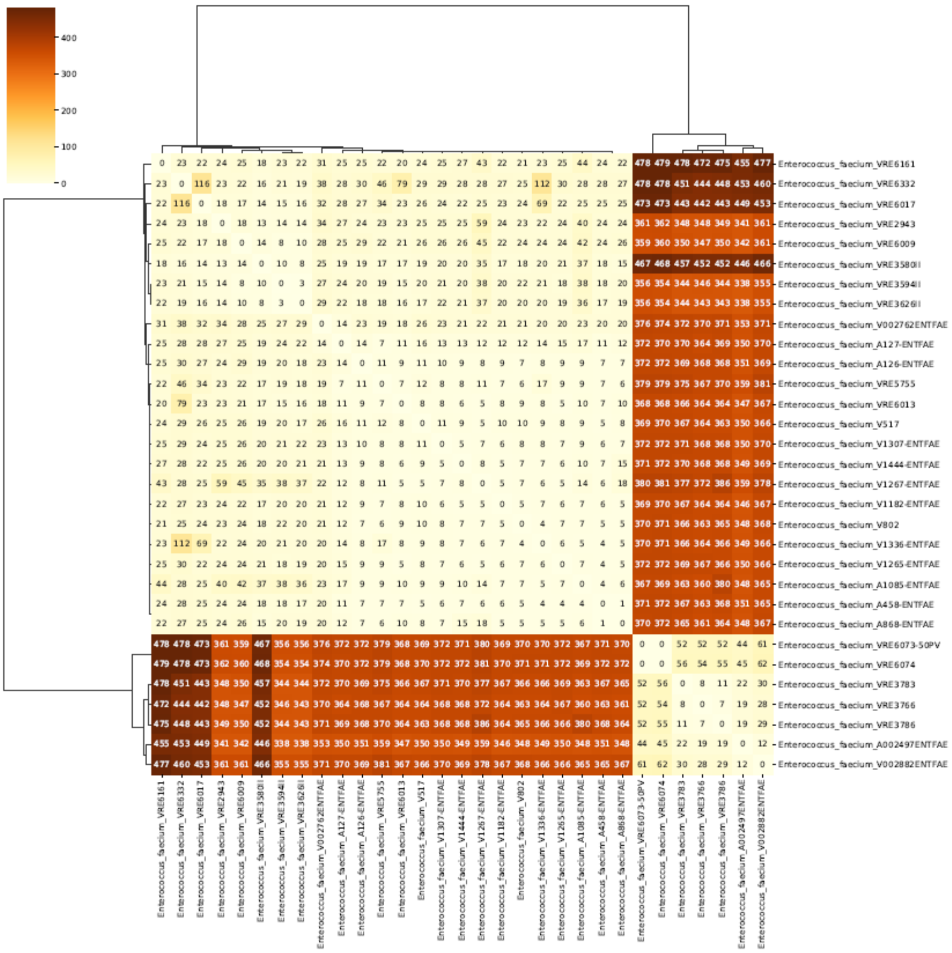
400
300
200
100
0
Enterococcus_faecium_VRE6161
Enterococcus_faecium_VRE6332
Enterococcus_faecium_VRE6017
Enterococcus_faecium_VRE2943
Enterococcus_faecium_VRE6009
Enterococcus_faecium_VRE3580II
Enterococcus_faecium_VRE3594II
Enterococcus_faecium_VRE3626II
Enterococcus_faecium_V002762ENTFAE
Enterococcus_faecium_A127-ENTFAE
Enterococcus_faecium_A126-ENTFAE
Enterococcus_faecium_VRE5755
Enterococcus_faecium_VRE6013
Enterococcus_faecium_V517
Enterococcus_faecium_V1307-ENTFAE
Enterococcus_faecium_V1444-ENTFAE
Enterococcus_faecium_V1267-ENTFAE
Enterococcus_faecium_V1182-ENTFAE
Enterococcus_faecium_V802
Enterococcus_faecium_V1336-ENTFAE
Enterococcus_faecium_V1265-ENTFAE
Enterococcus_faecium_A1085-ENTFAE
Enterococcus_faecium_A458-ENTFAE
Enterococcus_faecium_A868-ENTFAE
Enterococcus_faecium_VRE6073-50PV
Enterococcus_faecium_VRE6074
Enterococcus_faecium_VRE3783
Enterococcus_faecium_VRE3766
Enterococcus_faecium_VRE3786
Enterococcus_faecium_A002497ENTFAE
Enterococcus_faecium_V002882ENTFAE

S8: SKA SNP distances

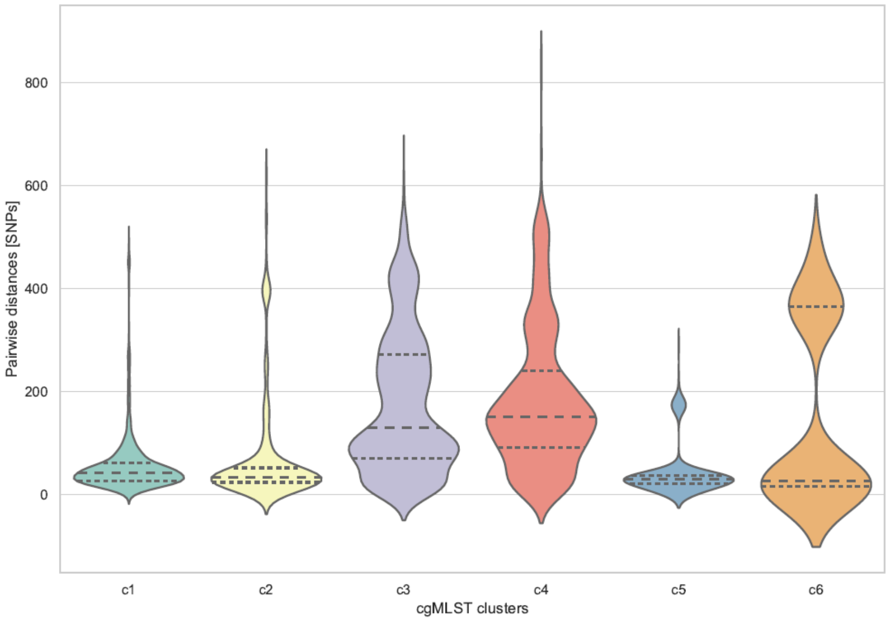

S9: Characteristics of the six largest standard cgMLST-clusters and results of additional genomic approaches, including standard cgMLST with threshold other than default settings (≥10 allels and ≥3 allels difference for attribution of clusters, respectively)

**Standard cgMLST threshold 20**		**Sus-pected outbreaks**	**Adhoc cgMLST threshold 20 allels**		**Standard cgMLST threshold 10 allels**		**Standard cgMLST threshold 3 allels**		**Core SNP**		**SKA**		**Van gene (N isolates)**				**Hospitals**		**Years**
Name	N iso-lates	N clusters	N clusters	Median isolates per cluster (min-max)	N clusters	Median isolates per cluster (min-max)	N clusters	Median isolates per cluster (min-max)	N clusters	Median isolates per cluster (min-max)	N clusters	Median isolates per cluster (min-max)	A	B	A+B	Not found	N Hospital Net-works	N Hospitals	N
**c1**	140	28	1	na ((139)	1	na ((136)	10	2,5 (2-83)	10	3,0 (2-54)	18	2,5 (2-8)	--	135	3	2	3	6	6
**c2**	100	21	2	50,0 (4-96)	2	na (4-96)	12	3,5 (2-31)	4	6,0 (2-63)	15	2,0 (2-9)	94	3	--	3	3	6	3
**c3**	95	20	6	3,5 (2-78)	5	3,0 (2-83)	12	2,5 (2-45)	11	4,0 (2-29)	12	2,0 (2-19)	86	3	--	6	3	5	4
**c4**	71	8	8	3,0 (2-42)	6	3,0 (2-43)	13	3,0 (2-16)	12	3,0 (2-13)	12	2,5 (2-16)	71	--	--	--	3	5	4
**c5**	38	5	2	19,0 (2-36)	1	na ((38)	6	5,5 (2-11)	4	6,5 (3-11)	5	5,0 (2-10)	38	--	--	--	2	5	2
**c6**	31	6	2	15,5 (7-24)	2	na (7-24)	4	4,0 (2-16)	3	3,0 (2-13)	4	2,0 (2-12)	30	--	--	1	2	5	6
